# Comparing fathers’ and mothers’ stunting knowledge and children’s oral health–related quality of life among stunted children

**DOI:** 10.1186/s12887-026-07495-x

**Published:** 2026-08-10

**Authors:** Arlette Suzy Setiawan, Fildza Ndarusasti, Hendriati Agustiani, Ratna Indriyanti, Netty Suryanti, Neti Juniarti, Laili Rahayuwati

**Affiliations:** 1 Department of Pediatric Dentistry, Faculty of Dentistry, Universitas Padjadjaran, Bandung, 40132 Indonesia; 2 Dentistry Undergraduate Program, Faculty of Dentistry, Universitas Padjadjaran, Bandung, 40132 Indonesia; 3 Department of Developmental Psychology, Faculty of Psychology, Universitas Padjadjaran, Sumedang, 45363 Indonesia; 4 Department of Community Dentistry, Faculty of Dentistry, Universitas Padjadjaran, Bandung, 40132 Indonesia; 5 Faculty of Nursing, Universitas Padjadjaran, Sumedang, 45363 Indonesia

**Keywords:** Stunting, Fathers, Mothers, Oral health–related quality of life, Parental perception, Children

## Abstract

**Background:**

Stunting is linked to negative oral health outcomes and can diminish children’s oral health–related quality of life (OHRQoL). Parental roles and perceptions, particularly differences between fathers and mothers, may influence how oral health impacts are recognized and managed. This study aimed to compare fathers’ and mothers’ stunting knowledge and perceptions of children’s OHRQoL among stunted children and to examine the association between parental stunting knowledge and OHRQoL.

**Methods:**

A cross-sectional survey was conducted in three subdistricts of Bandung City, Indonesia. Parents of stunted children aged 2–5 years were recruited during Posyandu sessions, followed by home visits to enroll fathers, yielding paired data from both parents of the same child. Stunting knowledge was assessed using a validated questionnaire adopted from prior research, and children’s OHRQoL was measured using the Parental-Caregiver Perceptions Questionnaire (P-CPQ). Data were analyzed using the Wilcoxon signed-rank test for paired comparisons and Spearman’s rank correlation for associations.

**Results:**

A total of 150 parent pairs were included. Fathers and mothers had comparable stunting knowledge scores (*p* = 0.112). Mothers reported significantly poorer OHRQoL than fathers (*p* < 0.001), with significant differences observed in the oral symptoms (*p* < 0.001), functional limitations (*p* = 0.026), and emotional well-being domains (*p* = 0.002). No significant difference was found in the social well-being domain (*p* = 0.229). Parental stunting knowledge was not significantly associated with children’s OHRQoL for either mothers (ρ = 0.144, *p* = 0.078) or fathers (ρ = 0.098, *p* = 0.232).

**Conclusion:**

Although fathers and mothers demonstrated similar levels of stunting knowledge, mothers perceived greater negative impacts of oral health on their children’s quality of life. Parental stunting knowledge was not associated with OHRQoL, underscoring the need for family-centered interventions that engage both parents and address caregiving practices and access to oral health services to improve the quality of life of stunted children.

## Introduction

Stunting is a chronic growth disorder characterized by impaired linear growth resulting from long-term nutritional inadequacy [1]. Long-term consequences of stunting include deficits in physical growth, as well as impaired mental, intellectual, and cognitive development [2, 3]. In Indonesia, data from the 2021 *Survei Status Gizi Balita Indonesia* (SSGBI) reported a stunting prevalence of 24.4%, affecting approximately 5.33 million children [4]. In the same year, Bandung city reported a decline in stunting by 1.34%, equivalent to 7.568 children, based on data from the Community-Based Nutrition Recording and Reporting System [5]. Previous research in Probolinggo Regency demonstrated a significant association between parental caregiving patterns and the incidence of stunting among young children, highlighting the importance of family-related factors in child growth and development [6, 7].

Stunting has been shown to affect various aspects of children’s oral health, including delayed eruption of deciduous teeth and an increased susceptibility to dental caries, partly due to alterations in salivary characteristics such as reduced flow rate and pH [8–12]. Oral diseases may negatively affect children’s physical comfort, functional ability, and emotional well-being, thereby impairing their overall quality of life [13]. The conceptual framework developed by Locker emphasizes the relationship between oral health and quality of life, commonly operationalized as Oral Health-Related Quality of Life (OHRQoL) [14]. Quality of life is defined as an individual’s perception of their position in life within their cultural and value context in relation to their goals, expectations, standards, and concerns [15]. Because children differ from adults in cognitive and emotional development, the assessment of OHRQoL in children requires specific measurement approaches. The Parental Caregiver Perception Questionnaire (P-CPQ), a component of the Child’s Oral Health Related Quality of Life (COHRQoL) instrument, is widely used to assess the impact of oral conditions on children’s well-being from the caregiver’s perspective 16].

Parental perception is formed through sensory input and cognitive interpretation of stimuli, which influence attitudes and behaviors toward child health [17]. Parents’ perceptions of their children’s oral health are known to shape preventive practices, such as supervising toothbrushing, determining appropriate timing and techniques for oral hygiene, and seeking regular dental care. These parental behaviors play a critical role in determining children’s oral health outcomes [18, 19]. However, caregiving responsibilities in many cultural contexts are still predominantly attributed to mothers, while fathers are often perceived mainly as economic providers. Despite this, growing evidence suggests that fathers’ involvement is essential in supporting children’s growth, development, and psychological well-being. Positive father–child relationships have been associated with better health-related behaviors and developmental outcomes in children [19, 20].

Parental knowledge of stunting may influence caregiving practices, nutrition-related decisions, hygiene behaviors, and the use of health services. In the context of oral health, such knowledge may shape how parents recognize children’s health problems, respond to oral symptoms, and seek preventive or curative care [21]. Therefore, differences in parental knowledge may contribute to differences in perceived oral health impacts and children’s OHRQoL.

Despite the recognized importance of parental roles, limited attention has been given to the specific contributions of fathers in relation to child oral health and stunting-related knowledge. Understanding differences in perceptions and knowledge between fathers and mothers is crucial for designing family-centered interventions for stunted children. Therefore, this study aimed to compare fathers’ and mothers’ stunting knowledge and perception of children’s oral health-related quality of life among stunted children. The study also aimed to examine the association between parental stunting knowledge and children’s OHRQoL.

## Material and method

### Study design and setting

This study employed a quantitative descriptive analytic design with a cross-sectional survey approach. This study was conducted in three subdistricts in Bandung City, Indonesia (Sukajadi, Antapani, and Coblong), which were designated as stunting loci by the Mayor of Bandung in 2021–2024. These areas were selected as part of the city’s stunting acceleration and prevention program.

### Participants and sampling

The study population consisted of parents (both mothers and mothers) of stunted children aged 2–5 years registered at the Sukagalih, Dago, and Griya Antapani health centers. Inclusion criteria were parents of children classified as stunted according to local health center records, and who agreed to participate. Parents of children older than 5 years, those with incomplete questionnaire responses, and those who declined participation were excluded.

Participants were recruited during the Posyandu session in selected subdistricts. Mothers of eligible stunted children were approached during Posyandu visits and invited to complete the questionnaire on site after providing informed consent. Subsequently, fathers of the same children were contacted and recruited through home visits conducted by the research team, during which the fathers completed the corresponding questionnaires. This procedure ensured paired data collection from both parents of the same child. A consecutive sampling approach was used during Posyandu sessions, followed by home visits to recruit fathers.

Data collection was conducted from December 2022 to July 2025. During the study period, 211 eligible stunted children aged 2–5 years were listed in the primary health center registries in the study areas. A total of 150 father-mother pairs were included in the final analysis, representing approximately 71% of the eligible population. Although a formal a priori power calculation was not performed, this sample was not performed, this sample was considered adequate because it covered a large proportions of the target population and allowed paired comparisons between fathers and mothers of the same child.

The extended recruitment period was required because the study aimed to obtain paired data from both fathers and mothers of the same stunted child. Mothers were recruited during Posyandu sessions, whereas fathers often required separate home visits because many were unavailable during Posyandu activities. Each parent pair was assessed once; therefore, the study retained a cross-sectional design. The same eligibility criteria, instruments, and data collection procedures were applied consistently throughout the study period.

### Variables and instruments

The variables in this study were parental stunting knowledge and children’s Oral Health–Related Quality of Life (OHRQoL) as perceived by parents. Two instruments were used. The first was the Parental-Caregiver Perceptions Questionnaire (P-CPQ), which was used to assess children’s OHRQoL based on parents’ perceptions of the impact of oral conditions on their children’s well-being. The P-CPQ consists of four domains: oral symptoms, functional limitations, emotional well-being, and social well-being. Parents were asked to report the frequency of their children’s experiences over the previous three months using a Likert-type scale [22]. Higher P-CPQ scores indicate poorer oral health-related quality of life.

The stunting knowledge questionnaire used in this study was adopted from the authors’ previous research. The instrument was developed based on relevant literature and national guidelines on stunting prevention and comprised five domains: theoretical knowledge, dietary prevention, hygiene-related prevention, health care utilization, and perceived impact of stunting. The questionnaire had undergone prior validation and reliability testing in the previous study. In the present study, the questionnaire was re-administered without modification. This questionnaire was based on the Guttman scale [22, 23].

The stunting knowledge questionnaire consisted of 28 dichotomous items covering five domains: theoretical knowledge, dietary prevention, hygiene-related prevention, health care utilization, and perceived impact of stunting. Example items included questions regarding the definition and causes of stunting, nutritional prevention, hygiene practices, use of health services, and long-term consequences of stunting on child growth and development. Responses were scored using a Guttman scale, with correct answers assigned a score of 1 and incorrect answers assigned a score of 0. The total possible score ranged from 0 to 28, with higher scores indicating better stunting knowledge. No categorical cut-off values were applied; therefore, the total score was analyzed as a continuous variable.

### Data analysis

Data were analyzed using SPSS (version 23). Descriptive statistics were used to summarize participants’ characteristics and study variables. Data distribution was assessed using the Shapiro–Wilk test. As the data were not normally distributed, continuous variables were presented as median and interquartile range (IQR).

Paired comparisons between fathers’ and mothers’ stunting knowledge and OHRQoL scores were performed using the Wilcoxon signed-rank test. Effect sizes were calculated using the formula r = Z/√N. Spearman’s rank correlation was used to examine the association between parental stunting knowledge and children’s OHRQoL scores. A *p*-value < 0.05 was considered statistically significant.

### Ethical considerations

This study was approved by the Research Ethics Committee of Universitas Padjadjaran, Bandung, Indonesia (No. 1309/UN6.KEP/EC/2022). The ethical approval was subsequently extended to cover the prolonged data collection period (December 2022–July 2025) under approval No. 855/UN6.KEP/EC/2024. Written informed consent was obtained from all participants prior to data collection.

## Results

### Participant characteristics

A total of 150 parent pairs (fathers and mothers of the same stunted child) were included in the final analysis. The sociodemographic characteristics of the participants are presented in Table 1. Most fathers and mothers were aged 21–35 years (58.7% and 60.7%, respectively), followed by those aged 36–45 years (33.3% of fathers and 34.7% of mothers). Only a small proportion of parents were younger than 20 years or older than 46 years.

**Table 1:** Sociodemographic Characteristics of Parents and Children (*N* = 150 parent pairs)

Variable	Fathers, *n* (%)	Mothers, *n* (%)
Parent age group		
* ≤* 20 years	1 (0.7%)	1 (0.7%)
21–35 years	88 (58.7%)	91 (60.7%)
36–45 years	50 (33.3%)	52 (34.7%)
* ≥* 46 years	11 (7.3%)	6 (4%)
Education level		
No formal education	0	0
Primary school	8 (5.3%)	8 (5.3%)
Middle school	8 (5.3%)	8 (5.3%)
High school	71 (47.3%)	81 (54.1%)
Higher education	63 (42.1%)	53 (35.3%)
Child’s age (years)		
Mean ± SD	3.21 *±* 0.954	
Median	3.00	

Regarding educational level, most fathers and mothers had completed high school (47.3% and 54.1%, respectively), followed by higher education (42.1% of fathers and 35.3% of mothers). The mean age of the children was 3.21 ± 0.95 years, with a median age of 3.0 years.

### Descriptive statistics of stunting knowledge and OHRQoL

Descriptive statistics of parental stunting knowledge and children’s oral health–related quality of life (OHRQoL) scores are shown in Table 2. The median stunting knowledge score was 14.0 (IQR 9.75–18.0) among fathers and 13.5 (IQR 10.0–18.25) among mothers. The median total OHRQoL score reported by both fathers and mothers was 3.0 (IQR 0.0–6.0), indicating generally low perceived impact on children’s OHRQoL.

**Table 2:** Descriptive statistics of parental stunting knowledge and children’s OHRQoL scores (Pair Fathers and Mothers)

Variable	Fathers	Mothers
Stunting knowledge score (0–28)		
Median (IQR)	14.00 (9.75-18.0)	13.50 (10.0-18.25)
Total OHRQoL Score (0–64)		
Median (IQR)	3.00 (0.0–6.0)	3.00 (0.0–6.0)
OHRQoL domains		
Oral symptoms (0–16), Median (IQR)	1.00 (0.0–2.0)	1.00 (0.0–3.0)
Functional limitation (0–16), Median (IQR)	0.00 (0.0–2.0)	0.00 (0.0–3.0)
Emotional well-being (0–12), Median (IQR)	0.00 (0.0–1.0)	0.00 (0.0–1.0)
Social well-being (0–20), Median (IQR)	0.00 (0-0.25*)	0.00 (0–1.0)

* The IQR of the father’s social domain is very narrow (P25 = 0; P75 ≈ 0.25)

Across OHRQoL domains, the median oral symptoms score was 1.0 for both parents, with a wider interquartile range among mothers. Median scores for functional limitations, emotional well-being, and social well-being were 0.0 in both fathers’ and mothers’ reports, suggesting minimal perceived impact in these domains for most children.

### Paired comparison between fathers and mothers

Paired comparisons between fathers’ and mothers’ reports are summarized in Table 3. There was no significant difference in stunting knowledge between fathers and mothers (Z = − 1.591, *p* = 0.112). In contrast, significant differences were observed in total OHRQoL scores (Z = − 5.369, *p* < 0.001), with a moderate-to-large effect size (*r* = 0.44).

**Table 3:** Paired comparison of fathers and mothers report on children’s OHRQoL and stunting knowledge (Wilcoxon Signed-Rank test, *n* = 150 pairs)

Outcome	Wilcoxon Z	*p*-value	Interpretation	Effect size (*r*)
Stunting knowledge	-1.591	0.112	Non-significant	0.13 (small)
Total OHRQoL	-5.369	< 0.001	Significant	0.44 (moderate-large)
Oral symptoms	-5.034	< 0.001	Significant	0.41 (moderate-large)
Functional limitation	-2.232	0.026	Significant	0.18 (small)
Emotional well-being	-3.169	0.002	Significant	0.26 (small-medium)
Social well-being	-1.203	0.229	Non-significant	0.10 (small)

At the domain level, mothers reported significantly higher OHRQoL scores than fathers in the oral symptoms (Z = − 5.034, *p* < 0.001; *r* = 0.41), functional limitations (Z = − 2.232, *p* = 0.026; *r* = 0.18), and emotional well-being domains (Z = − 3.169, *p* = 0.002; *r* = 0.26), indicating poorer perceived OHRQoL. No significant difference was observed in the social well-being domain (Z = − 1.203, *p* = 0.229).

### Correlation between stunting knowledge and OHRQoL

Spearman correlation analyses showed no significant association between parental stunting knowledge and children’s OHRQoL scores (Table 4). The correlation between mothers’ stunting knowledge and mothers’ OHRQoL reports was weak and not statistically significant (ρ = 0.144, *p* = 0.078). Similarly, no significant correlation was found between fathers’ stunting knowledge and fathers’ OHRQoL reports (ρ = 0.098, *p* = 0.232).

**Table 4:** Correlation between parental stunting knowledge and children’s OHRQoL

Pair of variables	Spearman’s rho	*p*-value	Interpretation
Mother knowledge vs. Mother OHRQoL	0.144	0.078	Non-significant (very weak)
Father knowledge vs. Father OHRQoL	0.098	0.232	Non-significant (very weak)

## Discussion

This study compared fathers’ and mothers’ stunting knowledge and their perceptions of children’s oral health–related quality of life (OHRQoL) among stunted children in urban areas of Bandung, Indonesia. The findings highlight important differences in parental perceptions of OHRQoL, while stunting knowledge did not significantly differ between fathers and mothers. In addition, parental stunting knowledge was not significantly associated with children’s OHRQoL, suggesting that knowledge alone may not translate into perceived oral health impacts in daily life.

Another possible explanation for the absence of association is that OHRQoL reflects subjective day-to-day experiences rather than knowledge itself [24]. Parents with adequate knowledge of stunting may still encounter practical barriers, including financial limitations, limited availability of pediatric dental services, competing caregiving responsibilities, and delayed treatment-seeking behaviors [25, 26]. Furthermore, because no clinical oral examination was conducted, it remains unclear whether parental perceptions accurately reflected the severity of children’s oral conditions.

Mothers reported significantly higher total OHRQoL scores than fathers, indicating a poorer perceived oral health–related quality of life among children when assessed by mothers. Significant differences were also observed in the oral symptoms, functional limitations, and emotional well-being domains, with moderate-to-large effect sizes for total OHRQoL and oral symptoms. This finding may reflect the greater involvement of mothers in daily childcare and health-related activities, which provides them with more frequent exposure to children’s oral symptoms and functional difficulties. Mothers may be more sensitive to subtle changes in their children’s comfort, eating difficulties, and emotional responses related to oral health problems [22, 27, 28]. In contrast, fathers’ comparatively lower OHRQoL scores may reflect less frequent day-to-day observation of oral health–related issues, rather than a true absence of problems [29–31].

Interestingly, no significant difference was found between fathers’ and mothers’ stunting knowledge scores. This suggests that public health messaging and community-based nutrition programs in the study areas may have reached both parents similarly. However, despite comparable levels of knowledge, mothers still reported greater impacts on OHRQoL. This discrepancy underscores the gap between knowledge and lived experience. While knowledge is necessary for appropriate health-related decision-making, it may not fully capture the experiential burden of caring for a child with oral health problems, which is more directly perceived by the primary caregiver [32].

The lack of significant correlation between parental stunting knowledge and children’s OHRQoL further supports the notion that knowledge alone may be insufficient to improve perceived child well-being [33]. Multiple contextual factors may mediate this relationship, including the severity of oral disease, access to dental services, caregiving burden, socioeconomic constraints, and parental coping strategies [34]. For example, parents with adequate knowledge may still face barriers to accessing timely dental care, resulting in persistent oral symptoms that negatively affect children’s quality of life [35]. Conversely, some parents with lower levels of formal knowledge may rely on experiential learning or informal support networks to manage their children’s oral health concerns [36].

From a public health perspective, these findings highlight the importance of family-centered approaches to stunting and oral health interventions. Programs that focus solely on improving parental knowledge may have a limited impact on children’s perceived quality of life if they do not address structural barriers to care and caregiving dynamics within families. Interventions that actively engage both fathers and mothers, promote shared caregiving responsibilities, and facilitate access to preventive and curative dental services may be more effective in improving children’s OHRQoL. In particular, empowering fathers to be more involved in routine oral health care and health-seeking behaviors could help align parental perceptions and potentially improve outcomes [33, 34].

This study has several limitations. The cross-sectional design precludes causal inference between parental knowledge and children’s OHRQoL. All measures were based on parental self-report, which may be subject to recall and social desirability biases. The study was conducted in selected urban stunting loci in Bandung City, which may limit the generalizability of the findings to rural settings or other regions with different sociodemographic characteristics.

A major limitation of this study is that no objective clinical oral health examination, such as dental caries assessment, was performed. Consequently, the relationship between perceived OHRQoL and actual oral health conditions could not be determined. This limitation may have influenced the interpretation of the association between parental knowledge and OHRQoL, as parental reports may not fully correspond to the clinical severity of oral disease.

Several potential confounding factors, including socioeconomic status, household income, family size, parental occupation, and access to dental care, were not included in the analysis. These factors may influence both parental knowledge and children’s OHRQoL and should therefore be considered in future studies. Another limitation is that no formal a priori sample size calculation was conducted. However, the final sample included 150 of 211 eligible parent pairs, representing a substantial proportion of the target population.

Selection bias may also have occurred because fathers were recruited through home visits and only those who were available and willing to participate were included. These fathers may have been more accessible or more engaged in family health matters than fathers who could not be contacted or declined participation.

Despite these limitations, this study provides novel insights into differences between fathers’ and mothers’ perceptions of children’s OHRQoL in the context of stunting. The findings underscore the need to incorporate both parents into oral health promotion and stunting prevention programs, while also addressing the broader social and health system factors that shape children’s quality of life. Future studies should consider longitudinal designs and the inclusion of clinical oral health assessments to further elucidate the pathways linking parental knowledge, caregiving practices, and children’s oral health–related quality of life.

## Conclusion

In this cross-sectional study of father–mother pairs of stunted children, fathers and mothers showed similar levels of stunting knowledge, but mothers reported poorer children’s OHRQoL than fathers, particularly in oral symptoms, functional limitations, and emotional well-being domains. Parental stunting knowledge was not significantly associated with perceived OHRQoL. These findings suggest that family-centered interventions should involve both parents and address not only knowledge but also caregiving practices, access to dental care, and contextual barriers. Because OHRQoL was assessed through parental perception without clinical oral examination, future studies should include objective oral health assessments and relevant socioeconomic variables.

## Data Availability

The datasets used and/or analysed during the current study are available from the corresponding author, Prof Arlette Suzy Setiawan (email: arlette.puspa@unpad.ac.id), on reasonable request.

## Abbreviations

OHRQoL: Oral Health–Related Quality of Life; Parental-Caregiver Perceptions Questionnaire (P-CPQ)
